# Activation of transforming growth factor-β_1 _and early atherosclerosis in systemic lupus erythematosus

**DOI:** 10.1186/ar1951

**Published:** 2006-04-28

**Authors:** Michelle Jackson, Yasmeen Ahmad, Ian N Bruce, Beatrice Coupes, Paul EC Brenchley

**Affiliations:** 1 Renal Research Laboratories, Manchester Institute of Nephrology and Transplantation, Manchester Royal Infirmary, Manchester, UK; 2 University of Manchester Rheumatism Research Centre, Central Manchester and Manchester Children's University NHS Trust, Manchester Royal Infirmary, Manchester, UK

## Abstract

The efficiency of activating latent transforming growth factor (TGF)-β_1 _in systemic lupus erythematosus (SLE) may control the balance between inflammation and fibrosis, modulating the disease phenotype. To test this hypothesis we studied the ability to activate TGF-β_1 _in SLE patients and control individuals within the context of inflammatory disease activity, cumulative organ damage and early atherosclerosis. An Activation Index (AI) for TGF-β_1 _was determined for 32 patients with SLE and 33 age-matched and sex-matched control individuals by quantifying the increase in active TGF-β_1 _under controlled standard conditions. Apoptosis in peripheral blood mononuclear cells was determined by fluorescence-activated cell sorting. Carotid artery intima-media thickness was measured using standard Doppler ultrasound. These measures were compared between patients and control individuals. In an analysis conducted in patients, we assessed the associations of these measures with SLE phenotype, including early atherosclerosis. Both intima-media thickness and TGF-β_1 _AI for SLE patients were within the normal range. There was a significant inverse association between TGF-β_1 _AI and levels of apoptosis in peripheral blood mononuclear cells after 24 hours in culture for both SLE patients and control individuals. Only in SLE patients was there a significant negative correlation between TGF-β_1 _AI and low-density lipoprotein cholesterol (*r *= -0.404; *P *= 0.022) and between TGF-β_1 _AI and carotid artery intima-media thickness (*r *= -0.587; *P *= 0.0004). A low AI was associated with irreversible damage (SLICC [Systemic Lupus International Collaborating Clinics] Damage Index ≥1) and was inversely correlated with disease duration. Intima-media thickness was significantly linked to total cholesterol (*r *= 0.371; *P *= 0.037). To conclude, in SLE low normal TGF-β_1 _activation was linked with increased lymphocyte apoptosis, irreversible organ damage, disease duration, calculated low-density lipoprotein levels and increased carotid IMT, and may contribute to the development of early atherosclerosis.

## Introduction

Transforming growth factor (TGF)-β_1 _is the most potent naturally occurring immunosuppressant [1]; it is produced by all cells of the immune system and plays a fundamental role in controlling proliferation and the fate of cells through apoptosis. In TGF-β_1 _knockout mice [2] lack of TGF-β_1 _initiates indiscriminate loss of self-tolerant T cells. Consequential dysregulation of B cell activity leads to production of systemic lupus erythematosus (SLE)-like autoantibodies [3] and development of a lupus-like illness, resulting in early death at 3–4 weeks [2]. Preliminary human studies suggest that TGF-β_1 _expression in SLE may be dysregulated. Production of TGF-β_1 _by lymphocytes isolated from SLE patients is reduced compared with that in control individuals [4]. Spontaneous polyclonal IgG and autoantibody production can be abrogated by treatment with interleukin-2 and TGF-β_1 _[5].

Atherosclerosis is a major cause of mortality and morbidity in SLE, with 6–10% of patients developing premature clinical coronary heart disease [6]. The 'protective cytokine hypothesis', recently reviewed [7], proposes that active TGF-β_1 _in the vascular wall is required to maintain the normal vascular wall structure and controls the balance between inflammation and extracellular matrix deposition in atherosclerosis. TGF-β_1 _is an inhibitor of smooth muscle and endothelial cell proliferation [8]. Mice heterozygous for the deletion of the TGF-β_1 _gene (tgfβ_1_^+/-^) have a 50% reduction in levels of TGF-β_1 _in artery walls and, when fed a cholesterol-enriched diet, such mice exhibit marked deposition of lipid in the artery wall as compared with wild-type mice [9]. In experimental models the evidence suggests that lack of TGF-β_1 _signalling promotes the development of atherosclerotic lesions and unstable plaques [10]. Therefore, because impairment in the TGF-β_1 _pathway has been associated with both an SLE-like illness and enhanced atherogenesis, we hypothesize that this pathway might represent a link between the inflammatory and atherosclerotic processes seen in SLE [11].

The aim of the present study was therefore to measure the efficiency of TGF-β_1 _activation in SLE, using a standard assay for active TGF-β_1 _in blood samples that were clotted under controlled conditions. We compared the level of physiological TGF-β_1 _activation during blood clotting in patients and control individuals, and examined whether TGF-β_1 _activation was associated with clinical phenotype, in particular inflammatory disease activity, cumulative organ damage and early atherosclerosis.

## Materials and methods

### Patients and control individuals

We recruited female Caucasian patients, of British descent, with SLE (1998 revised criteria) from clinics in the Manchester Royal Infirmary, North Manchester General Hospital and Blackburn Royal Infirmary. All studies in patients and control individuals were conducted with full informed consent of each participant. The study was approved by the North-West Multicenter Research Ethics Committee and the Scientific Advisory Board of the Wellcome Trust Clinical Research Facility. Patients underwent a full clinical assessment, including measurement of disease activity using the SLE Disease Activity Index (SLEDAI) [12]. Therapy was recorded, including current dose of steroids and antimalarial drugs. Damage was assessed using the American College of Rheumatology SLICC (Systemic Lupus International Collaborating Clinics) Damage Index (SDI) [13]. Healthy age-matched and sex-matched control individuals were recruited from the North West of England. In addition to the clinical assessment, current lipid and autoantibody profiles were noted. Following an overnight fast and avoidance of alcohol for 48 hours, 50 ml blood was drawn for laboratory studies. Specifically, antinuclear antibodies (ANAs), antibodies to double stranded (ds)DNA and anticardiolipin (ACL) were measured. ANAs were measured by indirect immunofluorescence on Hep2 cells. Antibodies to dsDNA (IgG) and cardiolipin (ACL; IgG and IgM) were detected using commercially available ELISAs (Aesku Diagnostics, Wendelsheim, Germany), with normal ranges of <25 units for anti-dsDNA antibodies and <16 units for ACL. C3 and C4 complement levels and the lupus anticoagulant were measured using the dilute Russell Viper Venom Test.

An ultracentrifugation method was used to remove very-low-density lipoprotein cholesterol from the plasma [14]. High-density lipoprotein (HDL)-cholesterol was determined following precipitation of low-density lipoprotein (LDL) from the resulting supernatant by heparin/Mn^2+ ^sulphate [14]. Total serum cholesterol, HDL-cholesterol and infranatant cholesterol were determined using the cholesterol oxidase: p-aminophenazone (CHOD-PAP) method. LDL-cholesterol was calculated as the difference between infranatant cholesterol and HDL-cholesterol. Serum triglycerides were determined by the glycerol phosphate oxidase: p-aminophenazone (GPO-PAP) method. LDL-cholesterol was calculated using the Friedewald formula (in mmol/l):

Calculated LDL-cholesterol = total cholesterol - HDL-cholesterol - (triglycerides/2.2)

### TGF-β_1 _activation assay

A variation of an ELISA format previously reported for detection of active TGF-β_1 _was employed [15,16]. Venous blood was collected without anticoagulant and 16 × 100 μl samples were immediately aliquoted into 2 × 8 well ELISA assay strips (Corning Plastics, Sigma-Aldrich Ltd, Poole, UK). One strip was immediately frozen at -20°C whereas the other was incubated at 37°C in a humidified incubator for 90 minutes and then frozen at -20°C. A solid-phase ELISA was carried out on stored paired samples of clotted (*n *= 8) and nonclotted blood (*n *= 8) arranged on the same plate. Plate lids with individual probes (TSP; Nunc, Fisher Scientific, Loughborough, UK) were coated over night with 100 μl/well of 2 μg/ml anti-TGF-β_1,2,3 _antibody (R&D Systems, Abingdon, UK) in coating buffer at 4°C in a humidified box. Lids were then washed in wash buffer (phosphate-buffered saline [PBS], 0.01% Tween 20 [Sigma-Aldrich Ltd, Poole, UK]), and blocked for 1 hour at room temperature with 150 μl/well ELISA buffer (PBS, 0.1% bovine serum albumen, 0.01% Tween 20). Samples were incubated at room temperature until just thawed and then the TSP lids were incubated in the samples for 2 hours at 4°C on a plate shaker. The lids were then washed and incubated with 100 μl/well of a 2.5 μg/ml anti-TGF-β_1 _antibody (R&D Systems) solution in ELISA buffer for 90 minutes at room temperature on a plate shaker. Lids were washed and incubated with 100 μl/well of a 1:20,000 dilution of donkey anti-chicken IgG antibody conjugated to peroxidase (Jackson Immunoresearch Laboratories, Stratech Scientific, Soham, UK) for 1 hour at room temperature. Freshly prepared 3,3',5,5'-tetramethylbenzidine substrate was used to develop the plate lids using 100 μl per well, the reaction was stopped by the addition of 50 μl per well of 2 mol/l H_2_SO_4_, and the plates were read at 450 nm. A relative Activation Index (AI) was calculated, by division of TGF-β_1 _levels in blood (A_450_) incubated at 37°C for 90 minutes by TGF-β_1 _levels in blood (A_450_) immediately frozen. The samples were collected, processed, stored and assayed in a standard manner with a between-batch variation (coefficient of variation) of ± 5%.

### Measurements of apoptosis

Apoptosis was measured in cells immediately following isolation of peripheral blood mononuclear cells (PBMCs) from blood, and after culture for 24 hours in 48-well plates (Nunc) at 1 × 10^6 ^cells per ml, 1 ml per well. Staining with annexin-V/propidium iodide (PI) identified early apoptotic cells. PBMCs were identified on the basis of light scatter properties. Dual colour histograms were analyzed for annexin-V/PI labelled cells and the percentages of apoptotic (fluorescein isothiocyanate [FITC]^+^PI^-^) and necrotic (FITC^+^PI^+^) cells determined. Cells in later stages of apoptosis were analyzed using PI staining to identify cells containing subdiploid amounts of DNA. Apoptosis of PI stained cells was defined as the percentage of cells with a fractional DNA content less than that in intact G_1 _cells (subdiploid cells).

For annexin-V/PI staining 1 × 10^6 ^cells were resuspended in 50 μl binding buffer (Roche Diagnostics, Lewes, UK) and incubated with annexin-V/FITC (Roche) and annexin-V/PI (Roche) for 15 minutes at room temperature in the dark. Samples were then washed once in PBS and resuspended in PBS, and immediate flow cytometric analysis was performed. Cells for PI staining were resuspended in 1 ml PBS, and 3 ml absolute ethanol was added whilst vortexing. Cells were fixed for at least 1 hour at 4°C. Following fixation cells were washed in PBS and resuspended in 1 ml staining buffer (50 μg/ml PI, 0.5 μg/ml RNase A, PBS). Samples were incubated at 4°C for 2 hours, washed once in PBS, resuspended in PBS and analyzed by flow cytometry. Flow cytometric analyses were performed on an Epics XL-MCL (Beckman-Coulter, High Wycombe, UK) flow cytometer. Ten thousand cells were analyzed for annexin-V/PI staining and 5,000 cells were analyzed for PI staining.

### Carotid artery intima-media thickness

All participants underwent a B-mode Doppler scan of their carotid arteries using a standard protocol. The common carotid artery (CCA) was scanned longitudinally and the intima-media thickness (IMT) was measured in the CCA, 1 cm proximal to the carotid bulb. IMT was the maximum distance between the intima-lumen and adventitia-media interfaces in areas without carotid plaque [17]. IMT was determined as the average of six measurements, three each from the left and right CCA. The intraclass correlation coefficient for IMT measurements, assessed in 15 participants on two separate occasions, 2 weeks apart, was 0.92 (95% confidence interval 0.84–1.00).

### Statistical analysis

TGF-β_1 _activation indices and lymphocyte apoptosis in SLE patients and control individuals were compared using the Mann-Whitney *U *test. Clinical associations were compared using the Mann-Whitney *U *test, and correlations were determined with the Pearson test. *P *< 0.05 was considered statistically significant.

## Results

### Clinical data from patients with SLE

We studied 32 patients and 33 control individuals with mean (± standard deviation) ages of 47.5 ± 9.4 years and 48 ± 10 years, respectively. SLE patients had a mean disease duration of 13 ± 5.8 years. The mean SLEDAI and SLICC damage scores were 1.75 ± 1.8 and 1.1 ± 1.2, respectively. Twenty-eight (91%) had a history of arthritis and 24 (75%) had mucocutaneous involvement. Three (9%) had a history of renal involvement. Twenty (60%) patients were receiving prednisolone (mean dose 3.6 ± 3.9 mg/day). Seventeen (53%) patients were receiving hydroxychloroquine and 18 (56%) patients were receiving other disease-modifying drugs. Seven (22%) were taking azathioprine, 5 (16%) methotrexate and one each were receiving one (3%) monthly pulse of intravenous cyclophosphamide and leflunomide. With regard to antibody profile at the time of study, 27 (90%) patients were positive for ANAs, 21 (69%) had elevated antibodies to dsDNA, 13 (41%) had ACL antibodies and nine (28%) had lupus anticoagulant.

### TGF-β_1 _activation index: an *in vitro *measure of the *in vivo *efficiency of TGF-β_1 _activation

To examine the ability of SLE patients and control individuals to activate TGF-β_1_, a ratio of levels of TGF-β_1 _in freshly collected blood and after 90 minutes of clotting at 37°C was determined. The degranulation of platelets during clotting results in release of excess latent TGF-β_1 _(estimated at >25 ng/ml [18]), some of which is subsequently activated physiologically over 90 minutes through protease action and interaction with thrombospondin, as occur in wound healing. That this initial pool of latent TGF-β_1 _was in excess is demonstrated by the lack of association between platelet count and TGF-β_1 _activation index (Pearson *r *= 0.143, *P *= 0.435; data not shown). Comparing levels of TGF-β_1 _in fresh unclotted blood and levels in blood clotted for a limited time (90 minutes) gives an indication of the overall efficiency of the TGF-β_1 _activation process in an individual. The TGF-β_1 _activation indices were similar between patients and control individuals (median [interquartile range] AI: 1.63 [1.31–1.88] in SLE patients versus 1.50 [1.26–1.73] in control individuals, *P *= 0.157; data not shown). The AI was determined once for each patient and control individuals on entry into the study. A study of the variation in TGF-β_1 _AI over time and with disease activity and treatment in SLE patients is beyond the scope of the present study.

### Apoptosis of peripheral blood mononuclear cells and TGF-β_1 _activation

**Figure 1:**
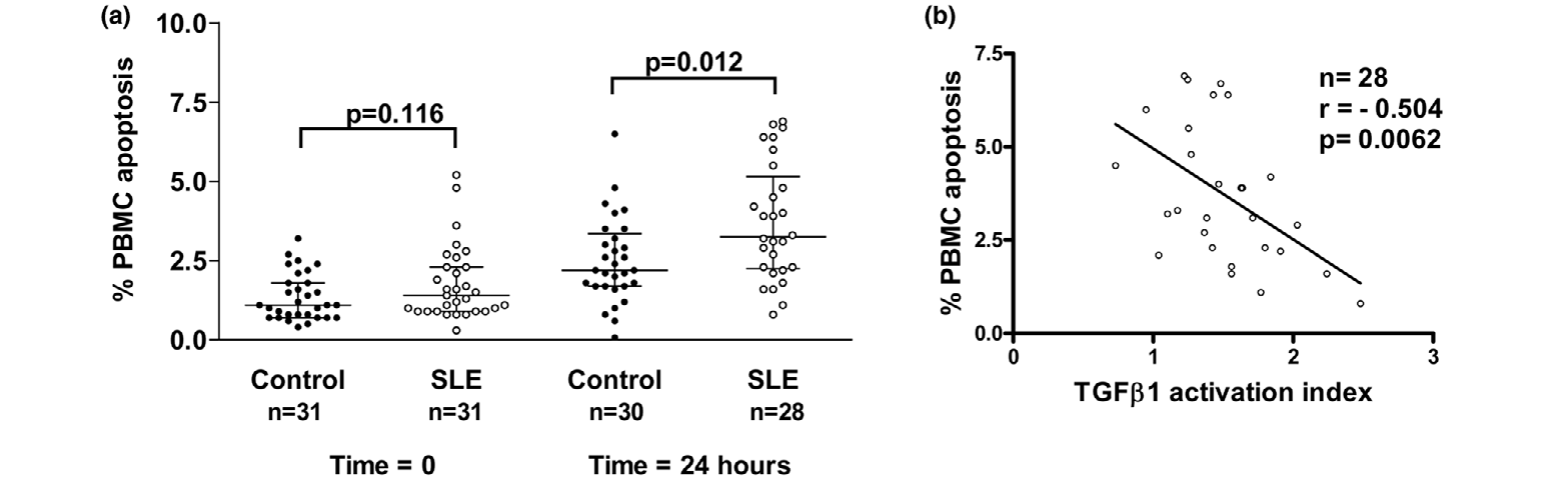
Association of apoptosis and TGF-β_1 _Activation Index. **(a) **Percentage of PBMCs undergoing apoptosis: control individuals versus SLE patients. Cells were stained with annexin-V and propidium iodide to differentiate early apoptotic cells from necrosing and late apoptotic cells. The thick horizontal bar denotes the median, and the whiskers show the interquartile range. Significance was calculated using the Mann-Whitney *U* test. **(b) **Degree of apoptosis in PBMCs from patients and TGF-β_1 _Activation Index are correlated, using Pearson test. PBMC, peripheral blood mononuclear cell; SLE, systemic lupus erythematosus; TGF, transforming growth factor.

SLE patients exhibited higher levels of apoptotic cells in the total PBMC population at 24 hours, as measured by annexin-V staining (median [interquartile range]: 3.25% [2.25–5.15%] versus 2.20% [1.7–3.35%], *P *= 0.012; Figure 1a). The TGF-βAI was significantly correlated with level of PBMC apoptosis at 24 hours in both control individuals and patients; Figure 1b shows the relationship for SLE patients (*r *= -0.504, *P *= 0.0062).

### TGF-β_1 _Activation Index and early atherosclerosis in control individuals and SLE patients

**Figure 2:**
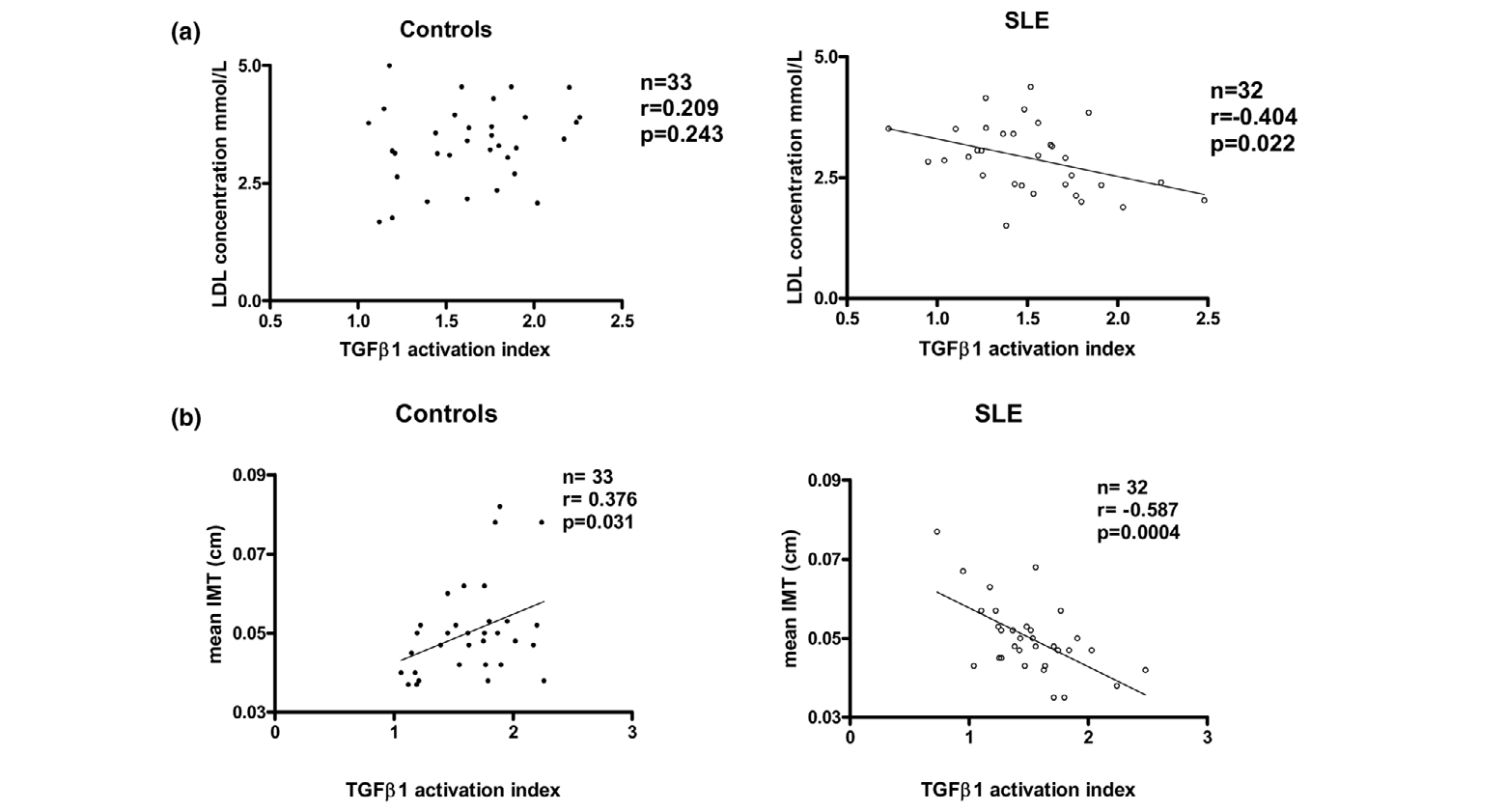
Correlation of LDL cholesterol and carotid IMT score with TGFβ_1 _Activation Index. **(a)**Correlation of LDL cholesterol and TGF-β_1 _Activation Index, using Pearson test. **(b) **Carotid artery IMT scores were correlated with TGF-β_1 _Activation Index for control patients and SLE patients, using Pearson correlation. A significant positive correlation was observed for control patients, whereas a significant inverse relationship was identified for SLE patients, with low TGF-β_1 _Activation Index being associated with increased mean carotid IMT score. Patients with SLICC score 0 have a higher TGF-β_1 _Activation Index than those with SLICC score 1–3, analyzed by Mann-Whitney *U* test. IMT, intima-media thickness; LDL, low-density lipoprotein; SLICC, Systemic Lupus International Collaborating Clinics; TGF, transforming growth factor.

There was no correlation between TGF-β_1 _AI and calculated LDL levels in control patients (Pearson *r *= 0.209, *P *= 0.243; Figure 2a), but there was a significant inverse correlation between the TGF-β_1 _AI and fasting LDL-cholesterol in patients with SLE (Pearson *r *= -0.404, *P *= 0.022; Figure 2a). TGF-β_1 _AI also correlated with total cholesterol in patients with SLE (Pearson *r *= -0.371, *P *= 0.037). TGF-β_1 _AI did not correlate with current steroid dose (*P *= 0.663), total duration of steroids (*P *= 0.986), dose of antimalarial (*P *= 0.589), disease-modifying antirheumatic drug therapy (*P *= 0.121), or SLEDAI (*P *= 0.913; data not shown).

**Figure 3:**
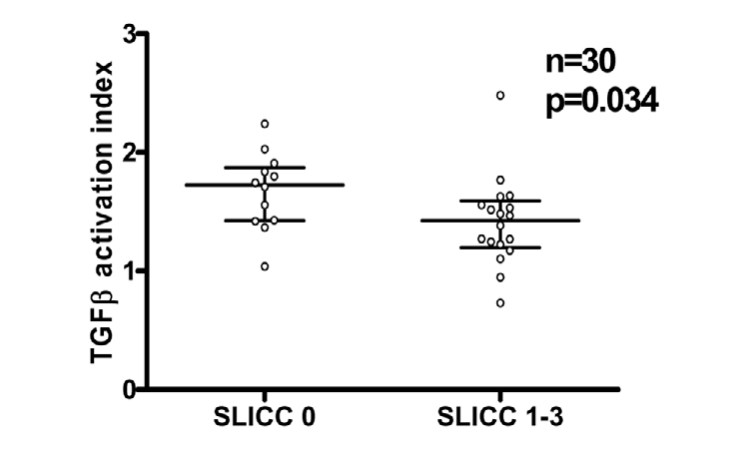
SLE disease severity and TGFβ_1 _Activation Index. Patients with SLICC score 1–3 have a significantly lower TGF-β_1 _AI than do patients with mild disease (SLICC score 0). SLICC, Systemic Lupus International Collaborating Clinics; TGF, transforming growth factor.

There was no difference in mean carotid IMT between patients and controls (mean ± standard error: 0.050 ± 0.002 cm versus 0.050 ± 0.002 cm; not significant). However, the correlation between TGF-β_1 _AI and carotid IMT in SLE patients and control individuals was qualitatively different (Figure 3a,b). In control individuals there was a significant positive correlation (*r *= 0.376, *P *= 0.031). In contrast, there was a highly significant inverse correlation in SLE patients (*r *= -0.587, *P *= 0.0004), such that low activation status was linked with higher IMT score. Analysis of covariance of IMT versus TGF-β_1 _activation and subject group, and testing the interaction term shows that the slopes in the control and SLE groups are significantly different (*P *= 0.0001). IMT exhibited a significant correlation with total cholesterol (Pearson *r *= 0.371, *P *= 0.037) but not with calculated LDL (Pearson *r *= 0.246, *P *= 0.175).

### TGF-β_1 _activation index, damage and disease duration

TGF-β_1 _activation was lower in patients with a SDI of 1 or greater (*n *= 18) than in those with a SDI of 0 (*n *= 12; median [interquartile range]: 1.43 [1.20–1.59] versus 1.73 [1.43–1.88], *P *= 0.034; Figure 3). The TGF-β_1 _AI in SLE patients inversely correlated with disease duration (Pearson *r *= -0.377, *P *= 0.033; data not shown).

## Discussion

We investigated the ability of SLE patients and control individuals to activate latent TGF-β_1 _in an *in vitro *assay that utilizes the physiological activation of latent TGF-β_1 _that occurs normally during blood clotting. The activation of TGF-β_1 _during clotting is complex, being mediated through several mechanisms [19,20] involving protease (plasmin) activation and interaction of TGF-β_1 _with thrombospondin-1. Using an ELISA assay validated for detection of active TGF-β_1 _[15,16], we determined the increased active TGF-β_1 _after clotting a standard volume of blood at 37°C for 90 minutes relative to the nonclotted sample. Although no differences in mean values were observed between AIs of control individuals and SLE patients, we hypothesized that the level of biological variation in the SLE group could be used as a surrogate marker of the efficiency of activating latent TGF-β_1_. This would allow us to establish whether low or high TGF-β_1 _activation efficiency could be linked with known abnormalities in lymphocyte apoptosis and markers of early atherosclerosis.

In accordance with other studies [21-23], we found an increase in apoptosis in the PBMCs of SLE patients compared with control individuals following 24 hours in culture. We found a lower rate of apoptosis at 24 hours (median 3.35%) compared with that described by Emlen and coworkers [21] (mean 12%). However, our SLE patients have a low disease activity score (mean SLEDAI score 1.75) and low damage score (mean SLICC score 1.1). This is consistent with the finding reported by Emlen and coworkers of a significant positive correlation between disease severity (SLAM [Systemic Lupus Activity Measure] index) and rate of apoptosis.

There was no significant difference after 24 hours of culture between the levels of apoptosis in patients receiving and those not receiving steroids at the time of study. In both patients and control individuals we observed a significant inverse relationship between level of PBMC apoptosis and TGF-β_1 _activation index (low TGF-β_1 _AI linked with high level of apoptosis).

The significance of increased PBMC apoptosis in SLE is profound, possibly reflecting increased levels of cells undergoing activated induced cell death and/or a defect in non-inflammatory phagocytosis of apoptotic cells. Failure to achieve programmed cell death and to clear apoptotic cell fragments could be a key pathogenic factor in the development of autoimmunity. As demonstrated in TGF-β_1 _knockout mouse, a loss of control in apoptosis affects the development and control of tolerance. Lack of TGF-β_1 _leads to increases in the levels of both the number of activated T cells and the levels of apoptosis in activated T cells and self-tolerant T cells – a situation that may be similar to that found in SLE patients. In the present study we report, for the first time, a significant association between ability to activate TGF-β_1 _and the degree of PBMC apoptosis at 24 hours. In the TGF-β_1 _knockout mouse there is an increase in mitochondrial membrane potential, and such increases are associated with initiation of apoptosis. It was recently demonstrated that the mitochondrial membrane potential in SLE patients is also increased [24]. Those SLE patients with low TGF-β_1 _AI status/increased apoptosis may be most at risk for the fundamental inflammatory process that drives SLE autoantibody production.

It is now well established that SLE patients are at fivefold to ten-fold increased risk for coronary heart disease compared with the general population. Classic risk factors have been found to be of importance in promoting the development of atherosclerosis in SLE [6]. However, after adjusting for Framingham risk factors, a significant excess risk remains [25]. This suggests that additional factors contribute to atherogenesis in SLE. Additional factors at play in SLE may include other metabolic changes such as renal impairment and homocysteine as well as adverse effects of steroid therapy and factors related to the underlying disease process, such as endothelial dysfunction and immune complex deposition [26].

The inverse correlation of TGF-β_1 _activation status and LDL-cholesterol levels identified in patients but not in control individuals is therefore highly relevant to this inflammatory process in the vascular wall. Two potential mechanisms whereby LDL might reduce TGF-β_1 _function have been described. First, it has been shown that very-low-density lipoprotein and LDL can inhibit the binding of active TGF-β_1 _to the type II TGF-β receptor and thereby suppress signalling through the receptor [27]. Second, and with particular relevance to this study, oxidized LDL is reported to interact specifically with thrombospondin-1 and inhibit the thrombospondin-1 dependent activation of latent TGF-β_1 _[28]. In SLE, Nuttal and coworkers [29] noted that LDL-cholesterol was more likely to exist as small dense particles that are more prone to oxidation. Although we did not measure LDL particle size, this difference in the type of LDL present in patients and control individuals may explain our observation of an inverse correlation of TGF-β_1 _AI and LDL in SLE patients, which was not seen in control individuals.

In the present study carotid IMT itself was not different between patients and control individuals; this is consistent with the findings of other larger series of SLE patients. Indeed, Roman and coworkers [30] found lower carotid IMT in SLE. Low TGF-β_1 _activation was also strongly associated with increased carotid IMT, an early marker of atherosclerotic change. It has been proposed that low levels of active TGF-β_1 _in the artery wall, resulting from apolipoprotein(a) inhibition of plasminogen activation and failure to activate latent TGF-β_1 _through plasmin proteolysis, allows endothelial and smooth muscle cell proliferation, leading to intima-medial expansion [8,31]. In our study this relationship was observed only in SLE patients, and the slopes in the SLE and control groups were significantly different (*P *= 0.0001), suggesting that the TGF-β_1 _interaction with IMT in SLE patients is different from that in age-matched/sex-matched control individuals. Although the TGF-β_1 _AI did not differ significantly between patients and control individuals, in the context of SLE increased oxidized LDL may promote a low TGF-β_1 _milieu, permitting excessive cellular apoptosis and enhancing the propensity for atherogenesis. Further studies are now needed to explore this hypothesis and it may be that several different factors govern the progression of carotid IMT in SLE. Such prospective studies will be needed to explore the interaction between inflammation and early atherosclerosis in more detail both in patients and unaffected individuals.

The association of low TGF-β_1 _AI and disease duration suggests that prospective studies of patients might identify changes in TGF-β_1 _activation and lipoprotein subfractions over time that could influence the development of atherosclerosis in SLE. Some of the atherogenic risk associated with LDL, in particular oxidized LDL, could be mediated through modulation of the availability of active TGF-β_1 _in the vasculature.

This observation has wider applications for prospective monitoring of TGF-β_1 _activation, not only in SLE but generally in patients developing atherosclerosis and fibrosis (for example, chronic allograft rejection). Therapeutic manipulation of the levels of active TGF-β_1 _may offer a new perspective in controlling the expression of disease in patients with SLE.

## Conclusion

Impairment of the TGF-β_1 _system in SLE not only may impact on the autoimmune pathophysiology of the disease but also may modulate the development of atherosclerosis and the increased risk for cardiovascular disease. Low activation of TGF-β_1 _is associated with increased apoptosis of PBMCs, increased carotid IMT, high levels of LDL-cholesterol and more severe SLE disease score. The factors in blood that modulate activation of TGF-β_1 _remain obscure, but the link with LDL-cholesterol opens up a novel atherogenic pathway that requires further study.

## Abbreviations

ACL = anticardiolipin; AI = Activation Index; ANA = antinuclear antibody; CCA = common carotid artery; ds = double stranded; ELISA = enzyme-linked immunosorbent assay; FITC= fluorescein isothiocyanate; HDL = high-density lipoprotein; IMT = intima-media thickness; PBMC = peripheral blood mononuclear cell; PBS = phosphate-buffered saline; PI = propidium iodide; TGF = transforming growth factor; SDI = SLICC damage index; SLE = systemic lupus erythematosus; SLEDAI = SLE Disease Activity Index.

## Competing interests

The authors declare that they have no competing interests.

## Authors' contributions

MJ performed most of the laboratory assays, helped in statistical analysis of the data and helped to draft the manuscript. YA obtained consent from patients and collected the samples and patient data for the study, and participated in the coordination of the study and writing of the manuscript. IB conceived the study, selected the patients for study, participated in its design and coordination, and helped to draft the manuscript. BC developed and carried out the TGF-β activation assay, data analysis and contributed to the writing of the manuscript. PB conceived the study, participated in its design and coordination and data analysis and writing of the manuscript. All authors read and approved the final manuscript
